# SAStutorials.org – online tutorials on small-angle scattering data analysis

**DOI:** 10.1107/S1600576725001062

**Published:** 2025-02-28

**Authors:** Andreas Haahr Larsen, Jeppe Breum Jacobsen, Melissa Ann Graewert, Lau Blom Grøndahl, Carsten Svaneborg, Federica Sebastiani, Alexey G. Kikhney, Arwen Irene Ingrid Tyler, Shinji Kihara, Kristian Lytje, Jan Skov Pedersen, Neshat Moslehi, Ilja Voets, Bence Fehér, Viktor Holm-Janas, Jesper Bruun, Martin Cramer Pedersen, Jacob Judas Kain Kirkensgaard

**Affiliations:** a Department of Neuroscience University of Copenhagen Denmark; b Niels Bohr Institute University of Copenhagen Denmark; c European Molecular Biology Laboratory (EMBL) Hamburg Site Germany; d Department of Physics, Chemistry and Pharmacy University of Southern Denmark Denmark; e Department of Pharmacy University of Copenhagen Denmark; f Xenocs Nordic, Denmark; g School of Food Science and Nutrition University of Leeds United Kingdom; h Department of Chemistry and Interdisciplinary Nanoscience Center (iNANO) Aarhus University Denmark; i Department of Chemical Engineering and Chemistry Eindhoven University of Technology The Netherlands; j Department of Biophysics and Radiation Biology Semmelweis University Hungary; k Department of Science Education University of Copenhagen Denmark; l Department of Computer Science University of Copenhagen Denmark; m Department of Food Science University of Copenhagen Denmark; Argonne National Laboratory, USA

**Keywords:** small-angle scattering, SAXS, SANS, teaching, simulation, didactics, active learning, tutorials

## Abstract

Small-angle X-ray and neutron scattering (SAXS and SANS) are versatile techniques for studying the structure of various materials and particles, enabling investigations of structures from a few to hundreds of nanometres. However, interpreting SAXS or SANS data can be challenging, hence the need for effective training. Here, we present the website SAStutorials.org, which offers tutorials on small-angle scattering (SAS) data analysis. The website serves as a learning platform that supports active learning both in the classroom and through self-study. The tutorials cover basic concepts, advanced modelling and theoretical aspects of SAS. Each tutorial follows a structure based on the principle of SOLO taxonomy, guiding learners from minimal or no knowledge in a given area to the ability to tackle real-world problems. SAStutorials.org has been developed as a community tool, providing tutorials that encompass a broad range of data analysis types, leveraging various programs and investigating different materials. Moreover, the website and all its data are open source, encouraging contributions from the community.

## Abstract 2

We present SAStutorials.org, a website that hosts online tutorials for small-angle scattering data analysis through active learning.

## 1. Introduction

**Figure 1:**
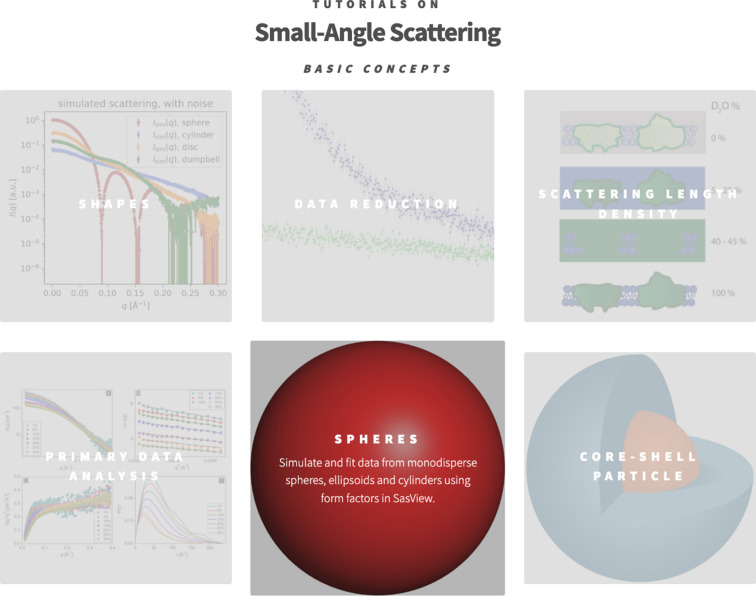
Screenshot from SAStutorials.org (January 2025), displaying the first few tutorials. When hovering the mouse over a given tutorial, a set of associated keywords appear.

We introduce SAStutorials.org (https://sastutorials.org), a website for learning how to analyze small-angle scattering (SAS) data (Fig. 1 ▸). Small-angle X-ray and neutron scattering (SAXS and SANS) are versatile experimental techniques that can probe structures ranging from a few to hundreds of nanometres. SAS is widely applied across scientific disciplines to investigate a broad range of materials, including solid energy materials (Povia *et al.*, 2018 ▸), synthetic nanoparticles (Li *et al.*, 2016 ▸), soft matter such as polymers or colloids (Lindner & Oberdisse, 2025 ▸; Narayanan, 2009 ▸), and biological macromolecules (Trewhella, 2022 ▸).

Unlike imaging techniques such as electron microscopy or super resolution microscopy, SAS does not provide real space images. Instead, the interpretation of SAS data often requires mathematical modelling. Moreover, SAS data often have limited information content, with only 10–30 free parameters (Glatter, 1980 ▸; Konarev & Svergun, 2015 ▸; Larsen & Pedersen, 2021 ▸; Moore, 1980 ▸; Pedersen *et al.*, 2014 ▸; Vestergaard & Hansen, 2006 ▸). Therefore, the analysis requires insight from scientists (*e.g.* about assumptions in various modelling approaches) to avoid misinterpretation of the data.

Research has shown that complex scientific concepts are often difficult for learners such as undergraduate and graduate students to use and interpret (Singh & Marshman, 2015 ▸). There is frequently a gap between students working with textbook problems and their encounters with subsequent real scientific problems; learners are often unable to apply knowledge acquired through solving textbook problems to real-life scientific inquiry (Modir *et al.*, 2019 ▸). Therefore, effective education is crucial for the field. This need is accentuated by growth in cross-disciplinary research in science (Glänzel & Debackere, 2022 ▸). In SAS, many groups are working with multiple experimental techniques. In such groups, even established scientists from different fields might not share the common ground needed to have productive discussions and make accurate interpretations (Mao *et al.*, 2019 ▸). These needs motivated the design of SAStutorials.org.

Though valuable resources such as university courses, international PhD schools and online platforms already exist (see *e.g.*https://SAStutorials.org/resources), we identified a need for a learning platform that is always accessible and is based on active learning. The tutorial webpage is inspired by MDtutorials.com (Lemkul, 2019 ▸), which contains tutorials on molecular dynamics simulations. This page is used extensively in the field of computational biology, with an estimated 250 000 non-unique annual users and over 1 000 000 page views (Justin Lemkul, personal communication, June 2024).

Our goal is that SAStutorials.org can be used in two learning situations. The first is MSc or PhD courses with SAS in their curriculum. We encourage teachers to use the tutorials or design new tutorials for lessons that support students’ active learning. Here, active learning is understood as a mode of instruction that emphasizes students’ increased levels of engagement with scientific phenomena, data, models and practices of the field (Lombardi *et al.*, 2021 ▸). The second learning situation is when students or researchers work with the tutorials without external supervision. To accommodate both learning situations, SAStutorials.org is constructed with some specific didactic principles in mind, which are detailed in this paper.

SAStutorials.org is founded on the philosophy of open science, and we offer it as an extendable community tool. This is why the webpage and the all data are available via GitHub (https://github.com/andreashlarsen/SAStutorials). Furthermore, teachers of SAS techniques can find guidelines on how to contribute a new tutorial at SAStutorials.org.

## 2. Tutorial design and didactical considerations

It is well established that active learning is effective (Deslauriers *et al.*, 2019 ▸; Freeman *et al.*, 2014 ▸; Theobald *et al.*, 2020 ▸), and therefore the tutorials promote student activation through tasks and challenges.

The design of SAStutorials is based on the Structure of Observed Learning Outcome (SOLO) taxonomy (Biggs & Collis, 1982 ▸), with a clear connection to real-world problems. SOLO describes learning as progression over five levels: (1) the ‘pre-structural’ level, where the learner has minimal knowledge about the subject or shows misunderstandings; (2) the ‘uni-structural’ level, where the learner shows understanding of a single aspect; (3) the ‘multi-structural’ level, where the learner can identify and work with several concepts but is not able to connect them; (4) the ‘relational’ level, where the learner can accurately connect concepts and identify patterns across concepts; and (5) the ‘extended abstract’ level, where the learner can apply the concepts and skills in novel ways, for example, in hypothesizing about or solving real-world problems. The SOLO taxonomy was developed to gauge the level of student understanding after a course. However, it can also be applied to design teaching materials that encourage learners to progress through its five levels of understanding, and this is our intention with SAStutorials.org. By basing successive tutorial tasks on the SOLO taxonomy, our aim is to avoid the gap between textbook examples and the learners’ encounters with real scientific problems.

### 2.1. Tutorial structure

In accordance with the didactical considerations above, each tutorial contains six sections: (i) learning outcomes, (ii) prerequisites, (iii) introductory remarks, (iv) tutorial subparts, (v) challenges and (vi) perspectives.

The *Learning outcomes* section provides students and teachers with an overview of what can be learned from the tutorial, so they can assess whether that tutorial is relevant for them. Further, the learning outcomes help the students to assess if they have achieved them. If used as part of a course, the learning outcomes can help the teacher to create constructive alignment (Biggs, 1996 ▸), *i.e.* consistency between learning outcomes, teaching format and course assessment.

The prerequisites section (named *Before you start*) provides guidance for students on what to prepare before completing the tutorial, including details on required programs and instructions for their installation. Additionally, this section indicates whether completing other tutorials beforehand would be beneficial.

The *Introductory remarks* section includes a short introduction to the concept in the given tutorial. It may contain some theory, although more complex theory should be introduced elsewhere, *e.g.* in complementary lectures or textbooks. In this section, the student is met at the pre-structural level, and the relevant context is provided.

The tutorial subparts (named *Part I*:…, *Part II*:… *etc.*) are the core of the tutorial. Each part covers a central aspect of the overall topic of the tutorial and guides the student through a specific type of analysis. The results are discussed, along with typical pitfalls or misinterpretations. In each subpart, a key concept is acquired, and thus each subpart reflects a uni-structural level in the SOLO taxonomy model. Each subpart is a combination of information, walkthrough analysis examples and closed tasks [*i.e.* tasks with a predefined question, method and answer (Tamir, 1989 ▸)]. Through working with all subparts, the students are helped towards reaching the multi-structural level. The intention is also that students acquire deeper learning, since the subparts are generally not parallel learning elements, but serial, and concepts from the first subparts are referenced or used in later subparts, following the idea of scaffolding in learning (Wood *et al.*, 1976 ▸).

The *Challenges* section contains tasks that are, in contrast to the subpart tasks, relatively open (Tamir, 1989 ▸), meaning the problem is defined but the method and answer are not. The challenges can be solved using the skillset that is acquired through the subparts of the tutorial but may also be solved in different ways. The challenges are meant to facilitate a transition into the relational level, as the student must consider and apply relevant methods from the subparts and often a combination is needed for each challenge. Most challenges include real data [*e.g.* from the SAS biological data bank (SASBDB) (Kikhney *et al.*, 2020 ▸; Valentini *et al.*, 2015 ▸)] or round-robin studies (Pauw *et al.*, 2023 ▸; Trewhella *et al.*, 2022 ▸, 2024 ▸). In the *Perspectives*[Sec sec3] section, applications from the literature are discussed and referenced for further reading, thus consolidating the relational level.

These tutorials are not intended to guide learners to the extended abstract level of the SOLO model, where the student expands the learned concepts and methods, applies them to their own problems, or combines them in novel ways. However, facilitated by SAStutorial.org, the student or researcher may continue working with the techniques and thereby reach the extended abstract level, through their own research or course projects (see also suggestions in Table 1 ▸).

### 2.2. Modularity

Another key concept for the tutorial design is modularity. Each tutorial is an independent educational resource. The exception is that some tutorials explicitly recommend that key concepts, which can be learned in other tutorials, are known. The modularity helps the tutorials to be versatile, and they can be adapted by the students as self-study or by the teacher as part of their course design. This modularity also makes it possible to contribute new tutorials, so the webpage can be kept up to date with novel state-of-the-art analysis methods.

### 2.3. Choice of software packages in the tutorials

The tutorials use external software for data analysis. There are often several suitable software packages to choose from for a given type of analysis. In the case of the *Primary data analysis* tutorial, alternative approaches are provided, but in most cases, the analysis is only demonstrated with one software package.

We used four criteria for choosing software. (1) Familiarity/expertise: in the development team, we have written tutorials using programs with which we had familiarity. However, we welcome contributions from experts who use other software packages. (2) Open science: when possible, we have used open-source programs, following principles of open science. (3) Low installation threshold: when possible, we use programs with no installation (*i.e.* available through online servers). Alternatively, we use programs that are easy to install, over programs with dependencies that need to be installed from source. (4) Future-proof: where possible, we choose software that is regularly updated, preferably by a large user community. A prime example of this is *SasView* (https://sasview.org).

We welcome tutorials introducing any piece of software, as well as inclusion of guides on how an existing tutorial can be completed with alternative software packages (see the *Primary data analysis* tutorial).

Aside from analysis software, the tutorials utilize the online program *Shape2SAS* (Larsen *et al.*, 2023 ▸), which can be used to simulate SAS data from various shapes. This allows for effortless calculation and visualization of the scattering from particles with different shapes, sizes or contrast situations. The simulated data can further be exported and analysed as a virtual training experiment.

### 2.4. Recommendations for teachers: how to use SAStutorials.org in courses

To support students’ active learning, we generally recommend that teachers provide students with ample time to work through the tutorials, especially the challenges. Therefore, we recommend that lectures preceding student work be kept brief and to the point. This recommendation aligns with recent studies suggesting that student attention declines rapidly during lectures (Darnell & Krieg, 2019 ▸).

When students work with the tutorials, it is useful for the teacher to circulate the room and observe students’ work. The teacher can then engage with the student if needed, and provide hints and questions for reflection. During work with the challenges, students are expected to learn by actively connecting knowledge elements themselves (Ruiz-Martín & Bybee, 2022 ▸). Therefore, the teacher should be careful not to overexplain concepts and techniques.

We recommend that students get the opportunity to present their answers to challenges. This serves two purposes. First, the students can formulate their answers to other people, which helps consolidate their knowledge (Ruthven *et al.*, 2009 ▸). Second, the teacher can gauge student learning and provide feedback to help students achieve the learning outcomes as intended.

After their active work with a challenge, students are likely to be ready to hear more examples of how the technique is useful for research, or go in-depth with specific concepts and techniques. Thus, after active work can be a good place to have a lecture with such a focus.

Our recommendations are summarized in Table 1 ▸, which also provides ideas for variation and notes.

These recommendations are in line with teaching formats that usually outperform lecture-based formats, and we have received positive feedback from following these principles (see *Student feedback*[Sec sec2.8]). However, teachers should be aware that students might feel that they learn more from lecture-based formats, even if this is demonstrably false (Deslauriers *et al.*, 2019 ▸).

### 2.5. Recommendations for supervisors: SAStutorials.org for students in the laboratory

When a student wants to learn aspects of SAS data analysis, they can complete the tutorials at SAStutorials.org that are relevant to their scientific aims. Their supervisor may not necessarily be a SAS expert themselves, which makes it challenging to validate whether the student has learned what is outlined in the *Learning outcomes* section. To address this challenge, we provide a forum for discussing the tutorials via the GitHub page that also hosts the source code (https://SAStutorials.org/forum). This forum can be used to discuss tutorials, challenges or related questions about SAS in general.

### 2.6. Overview of the content at SAStutorials.org

As of January 2025, SAStutorials.org offers 16 tutorials that cover a wide range of topics. This includes ten tutorials on *Basic concepts*:

(1) *Shapes*: applying *Shape2SAS* (Larsen *et al.*, 2023 ▸) for generating shapes and to calculate the scattering from various particles.

(2) *Data reduction*: using the *ATSAS* package (Manalastas-Cantos *et al.*, 2021 ▸) for background subtraction, outlier detection *etc*.

(3) *Scattering length density*: calculation of scattering length densities, either manually or, for example, using the *SLD Calculator* in *SasView* (https://www.sasview.org).

(4) *Primary data analysis*: performing Guinier fits, making Kratky plots and applying indirect Fourier transformation to obtain the pair distance distribution using, for example, *Primus* from *ATSAS* or *BayesApp* (Hansen, 2012 ▸).

(5) *Spheres*: fitting simple homogeneous geometrical models to SAS data using *SasView*.

(6) *Core–shell particle*: fitting a core–shell model to SAS data using *SasView*.

(7) *Polydispersity*: determining size distributions for a collection of polydisperse spheres using *SasView* and determining free-form size distribution in *McSAS* (Pauw *et al.*, 2013 ▸).

(8) *Structure factor*s: modelling structure factors describing concentration and interacting effects using *SasView*.

(9) *Pair distance distribution*: generation and interpretation of pair distance distributions, including cases with interparticle interactions or inhomogeneous particles.

(10) *Liquid crystals*: indexing and identification of lyotropic liquid crystalline phases.

Additionally, four tutorials introduce *Advanced concepts and applications*:

(11) *Invisible detergents*: training in SANS contrast variation and use of partial deuteration.

(12) *Lamellar structures*: analysis and modelling of unilamellar and multilamellar lipid vesicles using *SasView*.

(13) *Comparing with electron microscopy*: comparison of SAXS data and electron microscopy density maps using *AUSAXS* (Lytje & Pedersen, 2024 ▸).

(14) *Simultaneous fitting*: analysis of SANS contrast variation data by performing simultaneous fitting using *SasView*.

Lastly, there are currently two tutorials on *Theory and derivations*:

(15) *Core–shell form factor*: deriving the mathematical expressions for the form factor of a core–shell particle.

(16) *Scattering equation builder (SEB)*: calculating form factors for polymers of interlinked subunits using *SEB* (Jarrett & Svaneborg, 2024 ▸).

In addition to the tutorials, the website features:

(1) Resources page (https://SAStutorials.org/resources): providing links to other tutorials, recorded lectures, community pages and reading materials on SAS.

(2) User discussion forum (https://SAStutorials.org/forum): facilitating discussions for SAS users and experts.

(3) Template for contributors: giving instructions on how to contribute new tutorials to the site.

### 2.7. Accessibility and continuity

We have made initiatives to ensure the accessibility, stability and continuation of the site.

(i) All materials, including data and html code, are available on GitHub (https://github.com/andreashlarsen/SAStutorials) using GitHub pages under the GNU public licence. The website’s availability and stability are therefore independent of the domain host or any university IT services. This solution is free of charge and has no time limit.

(ii) The domain https://sastutorials.org is secured and paid for until 2030, and this can be extended.

### 2.8. Student feedback

As of January 2025, SAStutorials.org has been used for nine MSc and PhD courses at University of Copenhagen, Roskilde University and Aarhus University. At our 2024 PhD summer school on SAS (https://indico.nbi.ku.dk/event/2069/), we aimed for a 50/50 distribution between lectures and hands-on exercises, using SAStutorials.org for the exercises. Student feedback was overwhelmingly positive: 80% found the balance between lectures and hands-on to be adequate, while 20% suggested an even greater emphasis on hands-on activities. Moreover, more than 80% of the students answered that they would likely (30%) or certainly (53%) use SAStutorials.org in their own projects. Usage numbers confirm this, with more than 1000 users and 5000 page views (as of January 2025) since its release in August 2024.

Aside from course evaluations, we encourage student feedback through the user discussion forum hosted on GitHub (https://SAStutorials.org/forum).

## 3. Discussion and perspectives

We believe SAStutorials.org provides a valuable educational tool for the SAS community. Additionally, we have gained some perspectives for improving the website further.

The website http://www.MDtutorials.com, which inspired this work, includes walkthroughs of simulations from selected research papers. This is likely contributing to its success (250 000 annual users), since the tutorials serve as detailed protocols with examples and explanations, which are used by researchers to conduct similar molecular dynamics simulations. The tutorial *Invisible detergents* at SAStutorials.org follows this idea, as it provides a walkthrough of a key concept from the paper by Midtgaard *et al.* (2018 ▸), namely, how to contrast match *n*-dodecyl-β-d-maltopyranoside micelles in SANS. This type of tutorial is particularly relevant for advanced modelling approaches, as it offers support for potential new users of a specific program or analysis protocol. Consequently, we welcome contributions for additional tutorials of this type.

Several techniques are closely related to ‘conventional’ SAS, including anomalous SAXS, grazing-incidence SAS, ultra-small-angle X-ray scattering and spin-echo SANS. These are not yet covered on SAStutorials.org, so tutorials on these techniques would be welcomed.

Self-study using the tutorials presents some challenges. One limitation is that the tutorials do not provide in-depth theoretical background. To address this, it could be beneficial to include additional information and links to reading material and recorded lectures, such that this information is available for self-study. Verification of learning outcomes during self-study is another challenge. This could potentially be alleviated using the GitHub forum for discussions. Moreover, we are currently designing a quiz-based system where feedback is provided to the students on the basis of their answers.

In summary, we have designed a set of online tutorials for basic and advanced analysis of SAS data, hosted at the website https://sastutorials.org. Each tutorial follows a scaffolding model, whereby the students are equipped with the appropriate skill set to perform a given type of analysis, including primary data analysis, fitting of form factors and structure factors, and design of contrast variation experiments. We plan to add more tutorials covering state-of-the-art analysis methods and encourage contributions. We hope the website will continue to grow as a community resource.

## Figures and Tables

**Table 1:** Suggested use of the tutorials from SAStutorials.org in courses

Before class	
(1)	Students read the relevant theory.

In class	
(1)	The teacher gives a lecture. Consider a short lecture that provides just enough information for students to start working with the tutorials.
(2)	Students work with the tutorial exercises. The teacher may assist.
(3)	The students formulate answers to the challenges. Answers can be presented through informal class discussion, mini-presentations (*e.g.* in next class) or a written assignment.
(4)	The teacher provides feedback to student work, for example, verbally in class after each student-formulated answer/mini presentation or via comments for written assignments.
(5)	The teacher shares research-based examples of use of the concepts and acquired skills, along with related challenges in the field.

Variations and notes	
(1)	Lectures in class can be replaced with reading material or a video lecture, following the idea of Flipped Classroom (Schell & Mazur, 2015 ▸).
(2)	Tutorial subparts can be completed as preparation before class, as these are self-explanatory and include hints.
(3)	In advanced MSc courses or PhD courses, students may describe and work on their own challenges and data, instead of those provided in the tutorials. This will help students reach the deepest level of the SOLO taxonomy model, the extended abstract phase, where the acquired skills are applied to tackle real-world problems, and methods are potentially combined or further developed.
(4)	Peer-to-peer feedback can be utilized instead of or in addition to teacher feedback when discussing the challenges (Hounsell & Hounsell, 2007 ▸; Voerman *et al.*, 2015 ▸).
(5)	Inspirational guest lecturers, such as collaborators or junior researchers in your laboratory, can provide research-based examples of the concepts in use.
